# Study of Prefabricated Crack Propagation on Monocrystalline Silicon Surfaces for Grinding Damage Analysis

**DOI:** 10.3390/ma17153852

**Published:** 2024-08-03

**Authors:** Bingyao Zhao, Ning Huang, Siyang Dai, Ping Zhou

**Affiliations:** State Key Laboratory of High-Performance Precision Manufacturing, Department of Mechanical Engineering, Dalian University of Technology, Dalian 116024, China; zby666666@mail.dlut.edu.cn (B.Z.); huangning@dlut.edu.cn (N.H.); daisiy@mail.dlut.edu.cn (S.D.)

**Keywords:** crack propagation, hard and brittle materials, grinding damage, prefabricated crack, fracture strength

## Abstract

Crack generation and propagation are critical aspects of grinding processes for hard and brittle materials. Despite extensive research, the impact of residual cracks from coarse grinding on the cracks generated during fine grinding remains unexplored. This study aims to bridge this gap by examining the propagation law of existing cracks under indentation using the extended finite element method. The results reveal that prefabricated cracks with depths less than the crack depth produced on an undamaged surface tend to extend further without surpassing the latter. Conversely, deeper prefabricated cracks do not exhibit significant expansion. A novel method combining indentation and prefabricated cracks with fracture strength tests is proposed to determine crack propagation. Silicon wafers with varying damaged surfaces are analyzed, and changes in fracture strength, measured by the ball-on-ring method, are utilized to determine crack propagation. The experimental results confirm the proposed crack evolution law, validated by damage assessments across different grinding processes, which is suitable for crack damage. The findings demonstrate that residual cracks from coarse grinding are negligible in predicting the maximum crack depth during fine grinding. This research provides a crucial foundation for optimizing the wafer thinning process in 3D stacked chip manufacturing, establishing that changes in fracture strength are a reliable indicator of crack propagation feasibility.

## 1. Introduction

Crack generation and propagation are focal points in the research concerning the machining process and performance analysis of hard and brittle materials. The material removal process during grinding is significantly influenced by crack propagation, with residual cracks playing a key role in subsequent process decisions [1,2,3,4,5]. The inherent low fracture toughness and extremely fragile properties of these materials require engineers to adopt a multi-step grinding process to strike a balance between efficiency and damage control requirements [6]. In semiconductor processing [7,8,9], a notable gap exists in the understanding of the evolution law governing coarse grinding damage during fine grinding. As a result, engineers often rely on empirical knowledge and databases, using several times the depth of coarse grinding damage as the removal allowance for fine grinding. This strategy aims to ensure that the damage depth is reduced to a stabilized value during fine grinding. However, this approach limits potential production efficiency improvements, particularly in 3D stacked chip manufacturing. If the expansion of rough grinding damage can be ignored in the fine grinding stage, the grinding efficiency will be greatly improved and the damage can be controlled. Therefore, maximizing grinding efficiency and enabling automatic process parameter decision making for hard and brittle materials requires an in-depth study of the propagation mechanism of existing surface damage under mechanical loading.

Research on grinding hard and brittle materials primarily focuses on subsurface crack damage. Numerous scholars have addressed the formulation of process parameters at various grinding stages and investigated their influence on damage. Evans et al. [10] carried out coarse and fine machining of different materials. During the fine grinding stage, multiple processing steps were implemented to minimize residual damage from coarse grinding. This intuitive approach aims to reduce the residual damage of coarse grinding, achieving a stabilized damage depth during fine grinding. In a study by Tonnellier et al. [11], a multi-step grinding process was explored for ULE and Zerodur materials. The results revealed damage depths of 12 μm during coarse grinding (utilizing a D76 diamond resin-based cup grinding wheel) and 3.5 μm during fine grinding (utilizing a D25 diamond resin-based cup grinding wheel). Notably, a removal amount of 50 μm, significantly exceeding the damage depth from coarse grinding, was used to ensure damage stability during fine grinding. Wang et al. [12] also adopted a similar processing method for discussion. Based on these collected experiences, Li et al. [13,14] designed a multi-step grinding experiment and processed different materials successively. According to the aforementioned research, a common practice to mitigate the influence of damage from previous processes involves substantial removal amounts to ensure experimental results’ stability. This precaution arises from the uncertainty surrounding the degree of propagation of residual damage from coarse grinding during the fine grinding stage.

The direct testing or prediction of subsurface damage during machining holds significant potential for meeting the requirements of process optimization and multi-step grinding processes. Therefore, several scholars have endeavored to predict grinding damage through theoretical models, sharing common underlying principles. The analysis begins with an examination of the abrasive cutting depth model, followed by a determination of the crack depth corresponding to the maximum cutting depth based on the indentation fracture mechanics. Zhou et al. [15] established a relationship between grit cutting depth and damage depth, demonstrating its validity through thinning tests on silicon wafers. Similarly, Zhang et al. [16] conducted related work, while Yin et al. [17] incorporated considerations of strain rate into their model for grinding depth and abrasive cutting depth of monocrystalline silicon, validated through experiment results. Some scholars have also explored the impact of wheel vibration [18] and grinding parameters [19], developing damage depth prediction models for various materials [20,21]. The existing literature reveals that grinding damage prediction is fundamentally based on the single-grit indentation damage model. However, whether using the classical indentation model [22] or the modified model [23,24,25,26], the influence of pre-existing cracks on the surface is not consistently considered. Even experimental studies on indentation damage depth have primarily focused on polished surfaces [27,28]. This prevailing research scenario has led to a continued reliance on removal amounts several times greater than the existing damage depth in actual production or research processes to ensure the stability of subsequent processing damage.

Understanding the crack propagation law is crucial for the accurate prediction of grinding damage and subsequent process optimization. Research on crack propagation has traditionally relied on Lawn’s fracture mechanics [29,30], with subsequent scholars further extending the basic model to accommodate the intricacies of the grinding process [31,32]. In recent years, there has been a growing focus on the influence of pre-existing damage on crack development using multiple scratches. Wang et al. [33] conducted two scratch tests on a silicon carbide surface with random abrasive grits on diamond wire saws. The study revealed that as the distance between the two scratches decreased, different types of cracks intersected and propagated to the surface, resulting in material detachment. Crack propagation was analyzed based on stress intensity factor calculations. Wang et al. [34] continued to study the interaction of lateral cracks under double scratches by a similar method. Feng et al. [35,36] studied the continuous scratching action of single abrasive grit and the simultaneous scratching actions of double abrasive grits on BK7 glass. The findings suggested that sequential action of two abrasive grits was more effective in minimizing subsurface damage than simultaneous actions on material surfaces. Yang et al. [37] found that under the interaction of multiple scratches, the critical depth of the cut was reduced, promoting lateral crack propagation. Jiang et al. [38] studied the crack initiation load and stress field of gallium nitride under two consecutive scratches. Despite the dedicated efforts of numerous scholars in this field, fundamental questions such as the conditions under which cracks propagate and the depth of their propagation under existing damage conditions remain unanswered.

This paper employs the extended finite element method to establish a prefabricated crack model using the indentation method. The study focuses on analyzing the propagation law of existing cracks at different positions and angles under indentation loading. The method involves combining indentation and prefabricated cracks with fracture strength tests to assess crack propagation. Specifically, cracks on the surfaces of various monocrystalline silicon are intentionally created through the indentation method, and the subsequent crack propagation is analyzed by monitoring changes in fracture strength. The experimental results validate the accuracy of the proposed crack evolution law. Further validation is provided by examining damage outcomes under different combinations of grinding processes. The feasibility of judging existing damage propagation methods is also demonstrated through grinding process experiments (both coarse and fine grinding are included). This integrated methodology, incorporating the extended finite element method, indentation, and fracture strength tests, contributes to a comprehensive understanding of crack behavior and offers a reliable means of assessing crack propagation in the context of various material processing scenarios.

## 2. Prefabricated Crack Simulation

In this section, the simulation of prefabricated cracks is explored to understand their behavior under different grinding conditions. The goal is to assess how residual cracks from coarse grinding influence damage during the fine grinding stage. By employing the extended finite element method (XFEM), the propagation of cracks in monocrystalline silicon is simulated, examining the effects of various parameters, including indentation depth, prefabricated crack depth, crack offset distance, and crack angles. This analysis provides a comprehensive view of crack behavior and aids in optimizing grinding processes for better damage control and efficiency.

The simulation model is first described, detailing the setup and innovations introduced in the approach. This is followed by an exploration of the material properties used in the simulations, focusing on the elastoplastic characteristics of monocrystalline silicon and the criteria for crack initiation and propagation. These insights are crucial for understanding the dynamics of crack propagation under realistic grinding conditions.

### 2.1. Simulation Model

**Figure 1:**
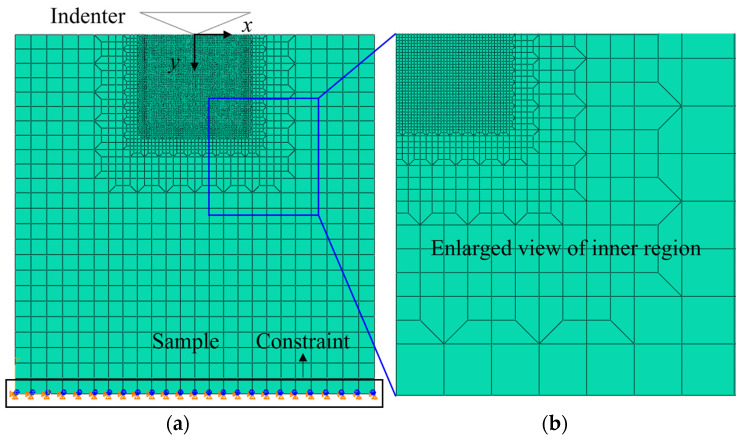
Indentation simulation model. (**a**) Simulation model. (**b**) Refined grid below indenter.

To study the impact of residual cracks from coarse grinding on damage during the fine grinding stage, the simulation of crack propagation in monocrystalline silicon with existing damage was conducted by the XFEM, as depicted in Figure 1a. The indenter was considered a rigid body with a central angle of 68°, moving along the y-direction, producing an indentation with a depth of 4–6 μm. The specimen, with a total of 13,396 nodes and dimensions of 160 μm × 160 μm, had refined meshing in the contact area between the indenter and the sample (0.4 μm × 0.4 μm), as illustrated in Figure 1b. The mesh size did not change when the simulation parameters changed. The friction coefficient between the indenter and the sample was 0.2. The bottom of the sample was constrained to move only in the horizontal direction. This model presents innovations compared to existing models in two aspects: (1) Previous models primarily focused on studying crack behavior on ideal surfaces. This study simulates crack propagation under various existing damage conditions, making it more representative of real grinding processes. (2) This model addresses the questions of whether cracks expand and what depth they reach after expansion in material with existing damage, providing valuable insights into the dynamics of crack propagation.

### 2.2. Material Properties

In this paper, the constitutive model of monocrystalline silicon was defined as elastoplasticity. Specifically, when the strain remained below the critical threshold for plastic deformation, the material was assumed to exhibit linear elasticity. Conversely, the material was assumed to transition into an elastoplastic state when the strain surpassed this critical threshold, as illustrated in Equation (1).

(1) $$\sigma =\left\{\begin{array}{ll} E\varepsilon & \varepsilon \leq \varepsilon_{Y}\\ \sigma_{Y}+E_{T}\left(\varepsilon -\varepsilon_{Y}\right) & \varepsilon \geq \varepsilon_{Y} \end{array}\right.$$

*ε*_Y_ is the critical strain of plastic deformation, and *σ*_Y_ is the corresponding critical stress. *E* is the elastic modulus, and *E*_T_ is the ratio of stress to strain in the plastic deformation stage. As per [39], the values assigned are *E* = 169 GPa and *E*_T_ = 79 GPa.

Crack initiation in this study followed the bilinear traction separation law [40]. The initiation of a crack occurred when the internal stress σ reached the critical value *σ*_c_. As loading progressed, the stiffness tensor degenerated along the softening branch. The formation of a crack took place when the separation displacement *δ* reached the critical value *δ*_c_. For linear softening, *σ* and *σ*_c_ were related to surface energy, which could be calculated using the fracture toughness and elastic modulus, as expressed in Equation (2). According to Reference [41], the yield limit of monocrystalline silicon is 6 GPa. In this model, it was assumed that fracture behavior occurred when the material reached the yield limit, and thus, this value was taken as the stress value (*σ*_c_) that initiates crack formation. The fracture energy (Γ) could be calculated according to Equation (2), with the calculated result being 3 J/m^2^.

(2) $$\Gamma =\frac{1}{2}\sigma_{c}\delta_{c}=\frac{(1-v^{2})K_{\mathrm{IC}}^{2}}{E}$$

where *E* is Young’s modulus, *v* is Poisson’s ratio, and *K*_IC_ is fracture toughness.

**Figure 2:**
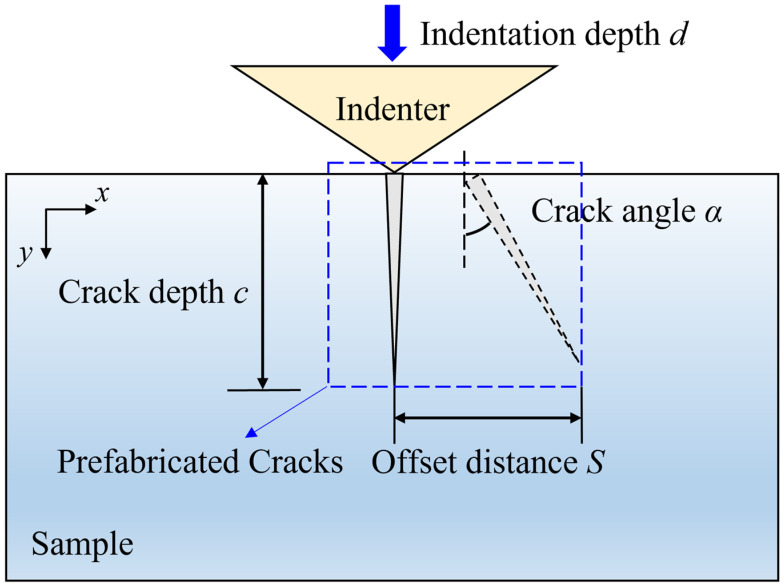
Schematic diagram of different prefabricated crack models.

According to [42], the morphology of distinct subsurface cracks resulting from various grinding conditions can be observed by cross-section microscopic observation. To closely approximate actual crack morphology, this study examines different indentation depths, various prefabricated crack depths, diverse crack offset distances, and different prefabricated crack angles tZo analyze crack propagation under existing damage conditions. Specific parameters are detailed in Table 1, and a schematic diagram is presented in Figure 2.

## 3. Experiments

This section presents a detailed account of the experimental procedures designed to investigate crack propagation in monocrystalline silicon. The experiments aim to understand how pre-existing damage from coarse grinding affects subsequent fine grinding stages. By prefabricating cracks and conducting fracture strength tests, the propagation behavior of these cracks under various conditions is assessed. The results provide insights into optimizing grinding processes for enhanced material integrity and efficiency.

### 3.1. Crack Propagation Law Test

#### 3.1.1. Prefabricating Cracks

To study crack propagation under the damaged surface of monocrystalline silicon, indentation tests with varying loads were carried out on both polished and ground wafers to prefabricate cracks. The ground wafer represented an already-damaged surface, serving as a sample for analyzing crack propagation under existing damage conditions. Meanwhile, the polished wafer acted as a comparative sample to assess whether crack propagation occurred under existing damage. Commercial electronic device-grade P-type (001) monocrystalline silicon wafers (Smart Card Co., Ltd., Shanghai, China) were used as the polished silicon wafers. The silicon wafers designated for grinding were processed using a self-rotating grinder (Okamoto, Japan, VG401 MKII) with a cup-type resin-bonded diamond grinding wheel (600#, abrasive particle size 24–28 μm). The sample size was 10 mm × 10 mm × 0.7 mm. Pre-crack tests were performed using a microhardness tester (Matsuzawa Co., Ltd., Akita, Japan, MMT-X3B, Vickers indenter). Indentation was performed at the center of the silicon wafer, with the diagonal of the indenter aligned parallel to the <110> crystal orientation of the monocrystalline silicon. The experimental scheme is detailed in Table 2. Subsurface damage (SSD) depth resulting from indentation was measured with a focused ion beam (FIB, Thermo Fisher Scientific, Inc., Waltham, MA, USA, Helios G4 UX).

#### 3.1.2. Fracture Strength Test

**Figure 3:**
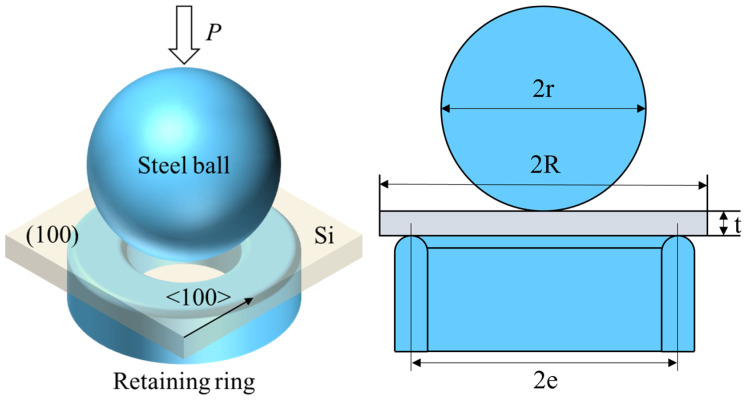
Ball-on-ring diagram.

Considering the limitations of damage detection methods such as the cross-sectional microscopic method [43,44], which hinder the intuitive judgment of crack expansion, this study investigates the crack propagation behavior of ground and polished wafers based on a fracture strength test. Specifically, changes in strength are used to determine whether the cracks have propagated. Under identical indentation loads, the ground wafer is considered to have existing damage to its surface, and its post-indentation strength is compared with that of the polished wafer to ascertain crack propagation. To mitigate the influence of edge defects on fracture strength [45], the ball-on-ring method was employed. This method provides insights into the relationship between damage depth and strength in the central region of the sample. By applying a single indentation solely in the central region, the influence of crack propagation on the fracture strength of the sample can be effectively assessed. The sample was placed on a circular support ring, and the spherical indenter descended to contact the sample, as shown in Figure 3.

When the indenter was in contact with the sample, an approximately uniform load was generated in the region of the central radius f, and the maximum radial stress at the center was considered equal to the tangential stress. This stress value can be obtained using Equation (3), proposed by Kirstein and Woolley [46]:

(3) $$\sigma_{\max}=\frac{3P(1+v)}{4\pi t^{2}}\left[1+2\ln\frac{e}{f}+\frac{\left(1-v\right)}{\left(1+v\right)}\left\{1-\frac{f^{2}}{2e^{2}}\right\}\frac{e^{2}}{R^{2}}\right]$$

where *P* is the load; 2*R* and *t* are the length and thickness of the specimen, respectively; 2*e* is the diameter of the hole; *f* is the size of the uniform load area; and *E* and *v* are the elastic modulus and Poisson’s ratio of the specimen, respectively.

In the fracture strength test, the diameters of the support ring and the indenter are specified as 8 mm and 10 mm, respectively. The elastic modulus of the monocrystalline silicon is 169 GPa, and the Poisson’s ratio is 0.28.

### 3.2. Grinding Process Test

To analyze the crack propagation on the already-damaged surface of monocrystalline silicon under different grinding combinations, a series of grinding process tests with different abrasive particle sizes (400# and 2000#, with particle sizes 30–40 μm and 6–8 μm, respectively), feed rates, and material removal thicknesses were designed, as outlined in Table 3. The monocrystalline silicon was processed by workpiece rotation grinding. The VG401 MKII ultra-precision grinding machine (Okamoto, Japan) was used. The grinding wheel was a cup-type resin-based diamond grinding wheel, with a diameter of 300 mm. The grinding wheel speed and workpiece speed were 2393 rpm and 144 rpm, respectively. Samples A–D underwent initial processing using process 1, with a material removal thickness of 300 μm. Subsequently, samples B–D underwent continued processing with parameter 2; the removal thicknesses were 3 μm, 5 μm, and 10 μm, respectively. Similarly, samples E–H were initially processed using parameters 1 and 2, with removal thicknesses of 300 μm and 50 μm, respectively. Samples F–H then underwent further processing with parameter 3, with removal thicknesses of 6 μm, 8 μm, and 10 μm, respectively. Each type of sample (Sample A–H) included three individual samples.

### 3.3. SSD Measurement

Cross-sectional microscopic observation was employed to measure the damage depth of ground single-crystal silicon samples A–H. The specific steps involved in the process were as follows: (1) The cross-section of ground monocrystalline silicon was polished with commercial colloidal silica slurry (COMPOL, FUJIMI Corporation, Osaka, Japan). The polishing parameters, detailed in Table 4, ensured the complete removal of damage caused by the previous process. (2) The polished sample was chemically etched with Young’s solution (H_2_O:49% HF:CrO_3_ = 50 mL:50 mL:7.5 g, 15 s) to fully expose the crack. (3) Following etching, ultrasonic cleaning with alcohol was performed. Finally, the sample section was observed with a confocal microscope (VK-X260K, Keyence, Osaka, Japan). To assess the impact of the deepest crack on subsequent polishing efficiency and sample strength, the 10 deepest cracks within a 10 mm range were counted.

## 4. Results and Discussion

In this section, the findings of the simulations and experiments are explored, focusing on the evolution of cracks under various conditions. The discussion is structured to provide insights into the behavior of cracks during indentation and grinding processes. Specifically, the propagation of prefabricated cracks, the influence of crack depth and inclination on fracture strength, and the verification of grinding processes are examined. These analyses aim to elucidate the complex interactions between indentation loads and pre-existing damage, thereby validating the proposed damage evolution law. By the end of this section, a comprehensive understanding of how different variables affect crack propagation and material integrity in monocrystalline silicon will be presented.

### 4.1. Crack Evolution

**Figure 4:**
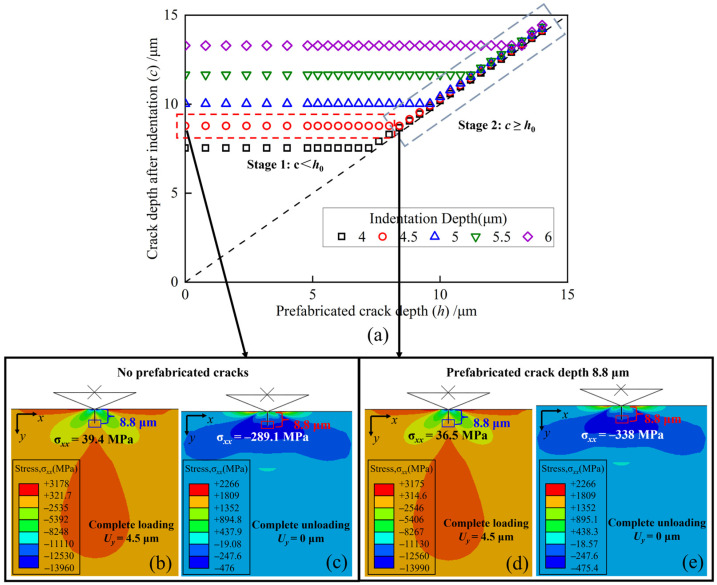
Simulation results encompassing diverse indentation depths and prefabricated crack depths. (**a**) Statistics of all simulation results. Complete (**b**) loading and (**c**) unloading scenarios without prefabricated cracks. Complete (**d**) loading and (**e**) unloading scenario with a prefabricated crack depth of 8.8 μm.

Figure 4a presents the simulation results for different indentation depths and prefabricated crack depths. The results reveal a bifurcation in the propagation of the existing damage, which occurs in two distinct stages. Compared with the crack depth *h*_0_ generated on the undamaged surface, observations indicate that at identical indentation depths, a prefabricated crack with a depth *c* less than *h*_0_ (*c* < *h*_0_) propagates during the indentation process. The indentation process refers to the process of the indenter pressing into the sample. Importantly, such propagation remains confined within the limits of *h*_0_. Conversely, when the prefabricated crack depth *c* equals or exceeds *h*_0_ (*c* ≥ *h*_0_), no discernible propagation occurs during indentation. Instead, the crack tip undergoes a slight downward displacement due to plastic deformation. 

To illustrate this in detail, the median crack propagation at an indentation depth of 4.5 μm is examined. Figure 4b,c depict the outcomes of full loading and unloading cycles in the absence of prefabricated cracks. Upon the initial contact of the indenter with the specimen surface, no crack initiation is observed. As the indentation depth increases and the stress reaches the maximum principal stress, the crack initiates and propagates downward until full loading is reached, as shown in Figure 4b. During the unloading phase, the median crack beneath the surface closes and recovers through elastic contact. While a residual stress zone remains on the surface, the stress conditions are not conducive to crack propagation, leading to the cessation of further expansion, as evidenced in Figure 4c. The entire indentation process aligns with Lawn’s description [22], validating the simulation’s accuracy.

Figure 4d,e depict the outcomes of complete loading and unloading cycles of the indenter when the prefabricated crack depth is 8.8 μm. These results reveal a lack of propagation in the prefabricated crack. A comparative analysis with results from Figure 4b,c shows tensile stress values of 39.4 MPa and 36.5 MPa at the tip of the crack under full loading and −289.1 MPa and −338 MP during full unloading. The final stress states in both conditions are approximately equal. The stress field distribution at the tip of the crack remains consistent with results observed under the undamaged surface, indicating a negligible secondary effect from abrasive grits on existing cracks. Consequently, with a prefabricated crack depth of 8.8 μm, the stress conditions at the tip do not meet the criteria for sustained expansion, resulting in the non-propagation of the crack.

**Figure 5:**
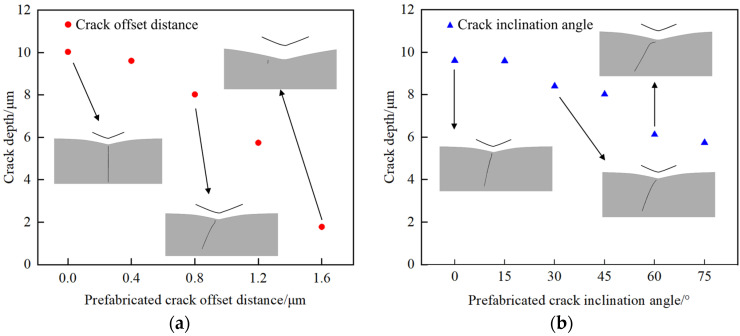
Simulation results. (**a**) Prefabricated cracks at different angles. (**b**) Crack offset distance at different angles.

This paper also explores the impact of prefabricated crack offset distance and angle on crack propagation. Figure 5a presents the propagation of a prefabricated crack at various offset distances. An increase in the offset distance correlates with a gradual reduction in the final depth of the extended crack. This phenomenon is attributed to the reduced impact of the indenter’s stress field on the prefabricated crack as the offset distance increases, resulting in a decreased final crack depth. 

Based on the above simulation, Figure 5b illustrates the results for prefabricated cracks inclined at angles ranging from 15–75° at an offset displacement of 0.4 μm. As the inclination angle increases, the depth of the extended crack decreases gradually. Both offset and inclined prefabricated cracks can alter the direction of crack propagation, indicating that the prefabricated crack angle and offset distance significantly influence the final crack depth. 

### 4.2. Fracture Strength to Determine Crack Propagation

#### 4.2.1. Subsurface Crack Depth

**Figure 6:**
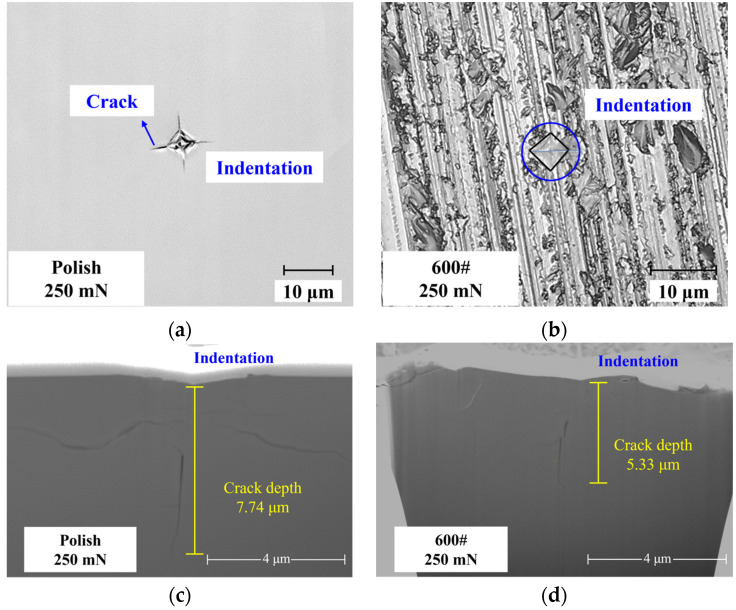
Prefabricated cracks made by the indentation method on different surfaces. (**a**) Prefabricated crack in a polished sample. (**b**) Prefabricated crack in a 600# ground sample. (**c**) Depth of damage in the polished sample revealed by FIB. (**d**) Depth of damage in the 600# ground sample revealed by FIB.

Figure 6a,b illustrate the indentation morphologies on both polished and ground surfaces under a 250 mN indentation load. Discernible cracks emerge on the polished surface under identical loading conditions, whereas the 600# ground surface, which shows grinding marks, exhibits no apparent cracks. The results regarding damage depth, measured by FIB after indentation on polished and ground samples, are presented in Figure 6c,d, respectively. The crack morphology is the standard median crack. These findings reveal a significant discrepancy in crack depth under the same indentation load. Specifically, the crack depth induced by indentation on the polished silicon wafer (with a non-destructive surface) surpasses that observed on the 600# ground silicon wafer (with existing damage on its surface).

**Figure 7:**
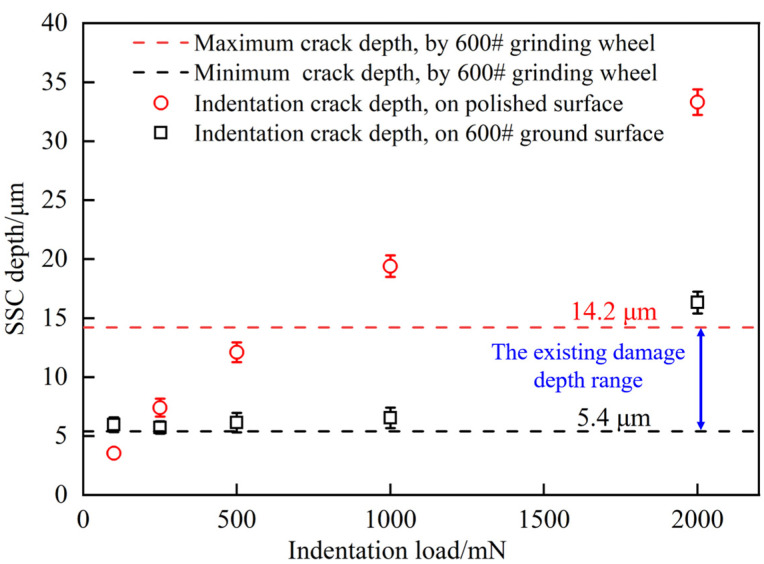
Subsurface crack depths of different damaged surfaces.

Figure 7 provides a comprehensive assessment of the damage depth for all polished and ground silicon wafers measured through FIB analysis. To ascertain the potential expansion of existing damage under the influence of indentation, it is crucial to acquire damage depth data for the 600# ground silicon wafer. Based on the work of Dai et al. [47], the distribution range of the 10 deepest crack depths observed in silicon wafers after grinding is shown in the dashed lines in Figure 7. The crack depth distribution ranges from 5.4 μm to 14.2 μm.

According to Figure 7, with an increase in indentation load, the crack depth on the polished surface gradually increases. In contrast, the subsurface damage depth of a 600# ground silicon wafer under indentation remains relatively constant. Except for the load *P* = 100 mN, the damage depth is consistently smaller than the indentation crack depth observed on a polished surface. When the load *P* < 1000 mN, the crack depth of the ground surface after indentation consistently falls within the documented damage depth range of the 600# ground silicon wafer. This demonstrated that, within this load range, the existing damage in the 600# ground silicon wafer exhibits negligible expansion following indentation. However, when the load *P* = 2000 mN, the crack depth of the ground silicon wafer after indentation surpasses the existing damage, indicating a significant expansion of the pre-existing damage. Moreover, it is hypothesized that the surface compressive stress induced by grinding may inhibit crack propagation, resulting in a crack depth less than that observed on the polished surface under the influence of indentation.

#### 4.2.2. Fracture Strength

**Figure 8:**
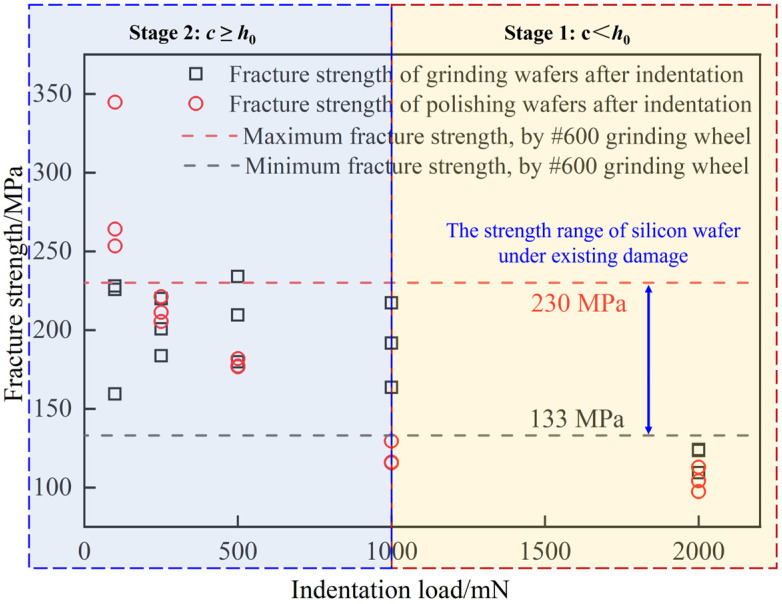
Fracture strength of surfaces with different damage statuses.

Previous research has identified a correlation between the fracture strength and the crack depth of single-crystal silicon [47]. Therefore, changes in fracture strength serve as an indicative of crack propagation. To provide a more straightforward and lucid analysis of crack propagation in a surface with existing damage, the fracture strength is used as an analytical parameter, as shown in Figure 8. 

For loads *P* = 100 mN, 250 mN, and 500 mN, the strength values observed in the ground wafer after indentation fall within the strength range of the grinding wafer. This indicates that the damage depth resulting from indentation on the ground silicon wafer remains within the damage depth range of the 600# ground silicon wafer. Consequently, within this load range, the existing damage in the 600# ground silicon wafer demonstrates minimal expansion. This situation is equivalent to the pre-crack depth *c* being greater than or equal to the damage depth *h*_0_ generated by the non-damaged surface, indicating negligible expansion of the existing crack. This substantiates the accuracy of the second stage in Section 4.1.

Under a load *P* = 1000 mN, the fracture strength of the ground sample after the indentation is higher than that of the polished sample after the indentation, which is also within the distribution range of the fracture strength of the grinding sample. It shows that the ground surface may inhibit the indentation to produce cracks or inhibit crack propagation [48].

At a load of *P* = 2000 mN, the fracture strength of the ground sample after indentation falls below the fracture strength range typical for the ground sample. This discrepancy indicates an expansion of existing damage. However, the depth of crack propagation does not exceed the depth of cracks generated under the non-damaged surface. This finding establishes that when the prefabricated crack depth (*c*) is less than the crack depth (*h*_0_) generated under the undamaged surface, crack expansion occurs to a certain extent but does not exceed the latter. This conforms to principles outlined in the first stage of Section 4.1. 

Overall, the results affirm that in the presence of pre-existing damage, when the prefabricated crack depth is less than the crack depth generated in an undamaged surface, crack expansion occurs but remains bounded by the latter. Conversely, when the prefabricated crack depth exceeds the non-damaged surface crack depth, significant expansion does not occur. This robustly validates the accuracy of the established laws governing the damage evolution, as discussed in Section 4.1.

### 4.3. Grinding Process Verification

**Figure 9:**
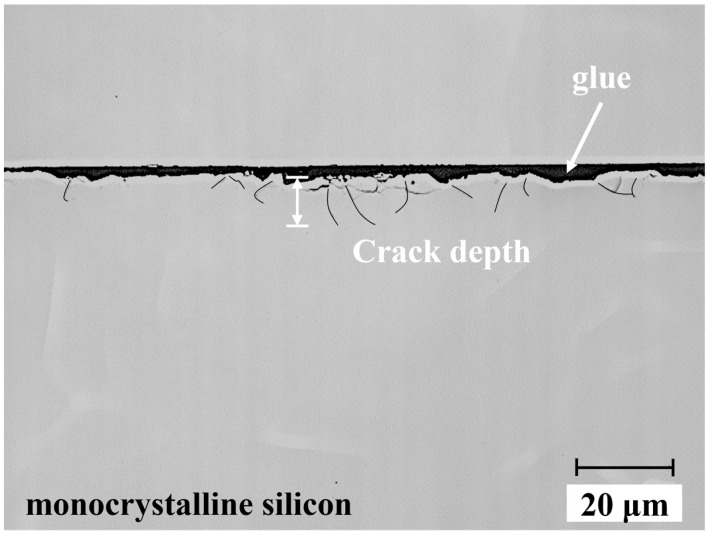
Damage detection in a ground monocrystalline silicon sample, with the total observation length spanning 10 mm. This image represents a singular snapshot within the observation path.

**Figure 10:**
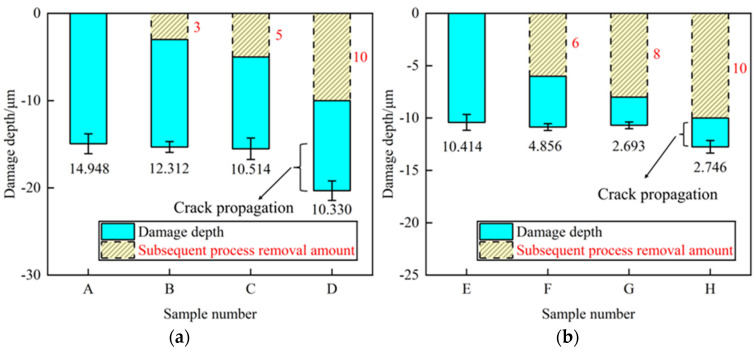
Damage evolution results under different grinding processes. (**a**) Damage evolution results in Samples A–D. (**b**) Damage evolution results in Samples E–H.

Figure 9 shows the damage depth in the monocrystalline silicon measured by the cross-sectional method, with the average damage depth of A–H presented in Figure 10a,b. The yellow histogram and red data denote the thickness of the removed material, while the blue histogram and black data represent the current damage depth. 

As depicted in Figure 10a, the damage depth of Samples B–C, processed by both process 1 and process 2, aligns with that of Sample A. The damage depth of sample A is the sum of the removal amount from process 2 and the corresponding damage depth. This observation indicates minimal expansion of cracks during this particular process. A similar situation is observed in Samples E–H, as shown in Figure 10b. When transitioning to parameter 3 from parameter 2, the sum of the initial removal amount and damage depth of Samples F and G is also similar to the damage depth generated by Sample E, indicating negligible crack expansion. This phenomenon shows that, in the presence of existing damage, when the prefabricated crack depth equals or exceeds the crack depth (*c* ≥ *h*_0_) generated in the undamaged surface, significant crack expansion does not occur—a result that is consistent with the second stage described in Section 4.1. 

Conversely, the sum of crack depth and removal amount of Samples D and G after process conversion exceeds the existing damage depth, validating crack extension. At this point, the prefabricated crack depth is less than the crack depth (*c* < *h*_0_) generated under the non-destructive surface but does not exceed it, congruent with the first stage described in Section 4.1. 

In the specific grinding test, a 400# grinding wheel was used in the coarse grinding stage, and a 2000# grinding wheel was used in the fine grinding stage. According to the grinding experiment results, Figure 10a,b verify the accuracy of the crack propagation law in the respective particle size range. This shows that the existing damage evolution law applies to each stage of grinding. The influence of abrasive particle size is qualitatively described in this paper, and the quantitative relationship between abrasive particle size and results will be further explored.

In summary, the diverse damage results from different grinding process tests provide further substantiation for the accuracy of the damage evolution law. Based on this law, substantial material removal during fine grinding is not imperative to ensure experimental results’ stability. This extends to the assertion that residual cracks from coarse grinding can be disregarded when predicting the maximum damage depth in the fine grinding stage.

According to the grinding test scheme in Section 3.2, sample E underwent rough grinding, while samples F–H underwent both rough and fine grinding. According to Figure 10b, the crack depth of sample E after rough grinding is 10.414 μm. The crack depths of samples F–H after rough grinding and fine grinding are 4.856 μm, 2.693 μm, and 2.746 μm respectively. The material removal thickness in the finely ground samples F–H are 6 μm, 8 μm, and 10 μm, respectively. The total depths are 10.856 μm, 10.693 μm, and 12.746 μm, respectively. Since samples F–H were processed with the same parameters as sample E in the coarse grinding stage, the crack depth generated by the coarse grinding is considered to be consistent with sample E. 

Comparing the total depth of samples F–H with the crack depth of sample E shows that samples F and G are similar, while sample H exceeds the crack depth of sample E. This proves that the cracks produced by the coarse grinding of samples F and G do not expand after fine grinding, while the cracks produced by the coarse grinding of sample H expand after fine grinding. This is consistent with the results obtained in Section 4.1 and Section 4.2. Thus, at the same indentation depth, the crack depth generated on the polished surface is *h*_0_. When the existing damage depth c exceeds *h*_0_, the crack does not expand significantly. In this case, the influence of cracks generated by rough grinding on the fine grinding stage can be avoided. In addition, the damage considered in this paper is mainly designated as crack damage; amorphous and dislocation damage generated in the fine grinding stage are not addressed.

## 5. Conclusions

This paper investigates crack propagation behavior on differently damaged surfaces of monocrystalline silicon, yielding several key conclusions:

1. Utilizing an extended finite element analysis, the crack propagation law for material with existing damage is elucidated. At a consistent indentation depth, if the prefabricated crack is shallower than the crack depth generated on the ideal surface, the crack tip stress field induced by the secondary action of the abrasive grits promotes the prefabricated crack, causing it to expand further but not exceed the latter. Conversely, when the prefabricated crack depth exceeds the crack depth generated on an undamaged surface, the secondary action of the abrasive grits has minimal impact on existing cracks, leading to negligible expansion.
2. A novel approach combining indentation, prefabricated cracks, and a fracture strength test is proposed to determine crack propagation. Analyzing the variation in fracture strength reveals that, at loads below 1000 mN, cracks in the ground indentation sample exhibit insignificant expansion. This methodology verifies the accuracy of the crack propagation law.
3. Through experiments involving different monocrystalline silicon grinding processes, it is observed that damage depth remains relatively constant during process transitions. This further validates the correctness of the existing crack propagation law under the secondary action of abrasive grits. The feasibility of assessing existing crack propagation is demonstrated by a combined utilization of the indentation method and fracture strength analysis. It also shows that the residual cracks from coarse grinding can be safely ignored when predicting the maximum damage depth in the fine grinding stage. This implies that substantial material removal is unnecessary to ensure the stability of the experimental results.

The findings presented in this research provide a pivotal foundation for optimizing and decision making in the multi-step wafer thinning process within the realm of 3D stacked chip manufacturing, where damage control is a critical goal. To optimize the process, maximize efficiency, and ensure the controllability of the damage, there remains a need for a predictive model of damage depth. In addition, the damage considered in this paper is mainly designated as crack damage. The amorphous and dislocation damage produced in the fine grinding stage are not included. Future research will focus on these two parts.

## Figures and Tables

**Table 1:** Simulation parameters.

Indentation Depth *d* (μm)	Prefabricated Crack Depth *c* (μm)	Offset Indenter Distance *S* (μm)	Crack Angle α (°)
4–6 Step size 0.5	0–14 Step size 0.4	0.4–1.6 Step size 0.4	15–75 Step size 15

**Table 2:** Indentation method prefabricated crack test scheme.

Sample	Indentation Load (mN)	Holding Time (s)	Total Number of Samples
Polished silicon wafer	100, 250, 500, 1000, 2000	15	15
#600 ground silicon wafer			

**Table 3:** Grinding experiment scheme.

Sample	Process 1			Process 2			Process 3		
	Abrasive Grit Size	Feed Speed (μm/s)	Removal Thickness (μm)	Abrasive Grit Size	Feed Speed (μm/s)	Removal Thickness (μm)	Abrasive Grit Size	Feed Speed (μm/s)	Removal Thickness (μm)
A	400#	5	300	-	-	-	-	-	-
B	400#	5	300	400#	1	3	-	-	-
C	400#	5	300	400#	1	5	-	-	-
D	400#	5	300	400#	1	10	-	-	-
E	400#	5	300	400#	1	50	-	-	-
F	400#	5	300	400#	1	50	2000	0.3	6
G	400#	5	300	400#	1	50	2000	0.3	8
H	400#	5	300	400#	1	50	2000	0.3	10

**Table 4:** Polishing parameters.

Average Particle Size (nm)	Polishing Plate Rotation Speed (r/min)	Workpiece Speed (r/min)	Polishing Pressure (psi)
72	300	50	1.8

## Data Availability

All data generated or analyzed during this study are included in the present article.
